# Cherry Gel Supplementation Does Not Attenuate Subjective Muscle Soreness or Alter Wellbeing Following a Match in a Team of Professional Rugby Union players: A Pilot Study

**DOI:** 10.3390/sports7040084

**Published:** 2019-04-05

**Authors:** Joe Kupusarevic, Kevin McShane, Tom Clifford

**Affiliations:** 1 Institute of Cellular Medicine, Newcastle University, Newcastle, NE2 4HH, UK; Joe.kupesarevic2@newcastle.ac.uk; 2 Newcastle Falcons R.F.C., Kingston Park, Newcastle NE13 8AF, UK; kevin.mcshane@newcastle-falcons.co.uk

**Keywords:** polyphenols, muscle pain, exercise recovery, rugby, intense exercise, anthocyanins

## Abstract

This study examined the effects of sour tart cherry juice (TC) on muscle soreness (MS) and wellbeing following a rugby union match in professional players. In a crossover design, 10 players from a senior squad in the top tier of England consumed either 2 × 30 mL servings of TC or an isocaloric cherry-flavoured control gel (CON) two days before, the day of, and two days following an 80 min match. Subjective wellbeing and MS were measured before the match (Pre), and for three days following the match (M+1, M+2, and M+3, respectively). MS was elevated from Pre at M+1 (CON, 111 ± 37 mm vs. TC 94 ± 41 mm) and M+2 (CON, 81 ± 35 mm vs. TC 72 ± 36 mm) (time effect; *p* = 0.0001; ηp^2^ = 0.821) but there were no differences between TC and CON at either time point post-exercise (*p* = 0.807; ηp^2^ = 0.035). Wellness scores were ~15% lower at M+1 (*p* = 0.023; ηp^2^ = 0.638) but there were no differences between the two conditions at any time point (*p* = 0.647; ηp^2^ = 0.160). In conclusion, tart cherry juice did not attenuate soreness or alter wellbeing in a team of professional rugby union players following a competitive match.

## 1. Introduction

Repetitive lengthening muscle contractions, like those associated with intense exercise such as sprinting, accelerating, decelerating, and resistance exercise, have been shown to cause muscle damage and initiate an acute phase inflammatory response [1,2]. The acute inflammatory response to exercise is complex but is generally characterised by an increased release of cytokines, which activate leukocytes—predominately neutrophils and macrophages—which then enter the affected areas to repair the damage [1,2,3,4,5]. While this response is ultimately beneficial for the remodelling of the affected tissues, these immune cells release toxic products such as reactive oxygen species that can also damage healthy cells and myofibrils [4,5]. For this reason, it is widely believed that the acute inflammatory response following strenuous exercise might exacerbate the existing muscle damage, and, thereby, attenuating the response could enhance the recovery process and reduce the severity of the associated symptoms, most notably, the loss of force generating capacity and feelings of muscle soreness (MS) [1,2,3,4,5]. 

Montmorency tart cherry juice (TC) supplementation has been touted as a supplement that might attenuate these symptoms following strenuous exercise. Indeed, a number of studies suggest that TC enhances the recovery of muscle function and attenuates MS following high-intensity exercise [3,6,7,8,9,10,11]. The main mechanism by which TC might expedite recovery is by attenuating the acute inflammatory response precipitated by the exercise bout [6,7,8,9,10,11]. Indeed, TC contains a number of phenolic compounds shown to have potent anti-inflammatory and antioxidant effects, most notably anthocyanins [9,11]. Support for these mechanisms of action is provided by the increasing number of studies in humans showing that TC reduces exercise-induced increases in interleukin (IL)-6 and c-reactive protein in the days following exercise [3,10,11].

Notwithstanding, these studies have all been conducted with untrained or sub-elite athletes, and in laboratory environments under controlled conditions (e.g., dietary restrictions and concomitant recovery aids banned) not reflective of real-world sport, questioning the ecological validity of the findings to date. As such, the aim of this pilot study was to assess the effects of TC supplementation on MS in a team of rugby union (RU) players following a competitive match. We were especially interested in RU because of the high-level muscle damage incurred after matches [12]. We focused on MS because the magnitude can be easily measured in this population, and rugby players report being sore for several days following a match [13].

## 2. Methods

### 2.1. Participants

Ten elite male RU players (age, 28 ± 4 years; height, 1.88 ± 0.64 m; mass, 106.8 ± 7.6 kg) from a team in the top league in England gave written informed consent for this study. Players completed a health screening questionnaire to check for relevant allergies and any other contraindications to the study procedures. Ethical approval was granted by Newcastle University Faculty of Medical Sciences Ethics board (registration number 01518). 

### 2.2. Experimental Design

This was a randomised, double-blind, placebo-controlled study. In a crossover fashion, participants ingested TC or an isocolaric placebo control (CON), twice-daily, before and after a competitive 80 min RU match. As this was a crossover design, players acted as their own control and completed both conditions (TC and CON). The randomisation was performed using an online generator (GraphPad Software, San Diego, CA, USA) by the senior author who was not involved in data collection. The 10 subjects were randomly assigned to consume either TC or CON first, which were labelled as A or B; for the second match, they consumed the opposing supplement. Supplementation began 2 days before the match and continued until the 3rd day after each match (spanning 5 days in total). Each dose was 30 mL and consumed in the morning and evening; we chose this dosing protocol because previous studies have shown benefits with TC when consumed in these amounts and when consumed both in the days before and after the exercise bout [6,7,8,9,10,11]. Furthermore, there is no data to suggest that a higher or lower dose would be more efficacious. However, unlike these studies, we were unable to start supplementation ≥4 days before the muscle-damaging bout, because the squad and starting line-up was not yet announced. We therefore started the supplementation 2 days before, when the starting team was announced, and we knew the players who would be playing the matches. Regardless, given that the main phenolic constituents in TC thought to beneficially affect inflammation and recovery (e.g., anthocyanins) are largely undetectable 8 h after consumption [14,15], we did not feel that having a shorter pre-load with the supplement would significantly impact our results, especially as no other studies have shown data to suggest a shorter pre-load period is less effective than a longer pre-load. Muscle soreness and wellbeing were assessed the day before the match and in the 3 mornings following (upon waking). The players wore GPS units (OptimEye S5B, Version 7.18; Catapult Innovations, Melbourne, Australia) during the matches to calculate external loads. 

Because of the nature of the participants, we could not control their post-exercise recovery procedures, that is, we could not prohibit them from using any *additional* recovery strategies after the matches, as is common in lab-based trials. Instead, we asked the players to replicate their recovery routine after each of the two conditions (e.g., TC and CON) by consuming the same whey protein drink and wearing the same compression garments, for the same amount of time. This was to ensure that any of the additional recovery strategies used were at least standardized between the two conditions. The players were instructed to maintain their habitual diet throughout. We did not collect food diaries because the author’s previous experience with this population has shown them to be unreliable and poorly completed. Nonetheless, as in previous studies [3,10,11], the players were instructed to follow a similar diet for the two matches, in an attempt to limit any influence that large deviations in dietary intake might have on the results. Thus, it is important to note that this study aimed to assess whether the addition of TC to their normal recovery routine and diet could help alleviate MS. 

### 2.3. Muscle Soreness

As in a previous study [16], players recorded their MS by drawing a vertical line on a 200 mm visual analogue scale (VAS) anchored by “no soreness” at 0 mm and “unbearably painful” at 200 mm. 

### 2.4. Daily Wellbeing

Daily wellbeing was assessed via a spreadsheet on the player’s mobile phones. Upon waking, all participants were required to log in to the app and rate their upper and lower body soreness, sleep hours and quality, mood, energy, motivation, and diet quality between 1 (poor) to 5 (excellent). The sum of these was used as an individual wellness score and for data analysis. 

### 2.5. Supplements

Each serving of the TC was 30 mL (kcal, 94; carbohydrates, 22 g; protein, 2 g) and contained cherry juice concentrate equivalent to 100 sour Montmorency cherries (*Prunus cerasus* L.) (Healthspan, Guernsey, UK). Akin to all previous studies with TC, because of its distinct taste, we could not precisely taste-match the CON. To get as close a match as possible, our CON was a gel that was similar in energy (kcal, 87; carbohydrate, 22 g; protein, 0 g), flavour, and texture (Science in Sport, 60 mL, GO Isotonic Energy, Cherry). These specific gels were tested because they were the closest taste-matched supplements available that had been batch tested for prohibited substances. Both were consumed as gels straight from sachets covered in opaque tape to blind the players from which gel they were receiving. To limit bias, players were told that both gels contained active cherry ingredients for recovery, but we were interested in the flavour they preferred. 

### 2.6. Data Analysis

All data are expressed as mean ± SD and statistical significance was set at *p* < 0.05. MS and wellbeing values were analysed using a repeated measures ANOVA with 2 treatment levels (TC vs. CON) and 4 repeated measures time points (PRE, match-day+1 (M+1), match-day+2 (M+2), match-day+3 (M+3). MS did not follow a normal distribution (*p* < 0.05 on the Kolmogorov–Smirnov test), so was log transformed prior to analysis. All GPS variables were analysed with paired student *t*-tests. IBM SPSS Statistics 23 for Windows (Surrey, UK) was used for data analysis. Partial-eta^2^ (ηp^2^) effect size statistics were considered either small (0.01–0.06), medium (0.06–0.14), or large (≥0.14) changes.

## 3. Results

There were no significant differences in external load measures between CON and TC, suggesting the physical demands of each match were similar (Table 1). 

**Figure 1:**
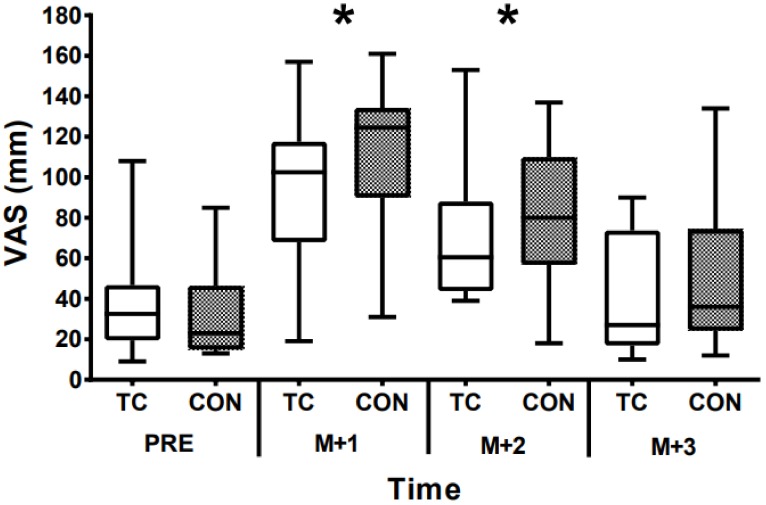
Muscle soreness, as measured by a visual analogue scale (VAS), before (PRE) and three days (match day+1, match day+2, match-day+3 (M+1; M+2, M+3, respectively)) after a rugby union match and control (CON) or tart cherry juice (TC) supplementation. * Denotes different PRE time point (*p* < 0.05) *n* = 10.

**Figure 2:**
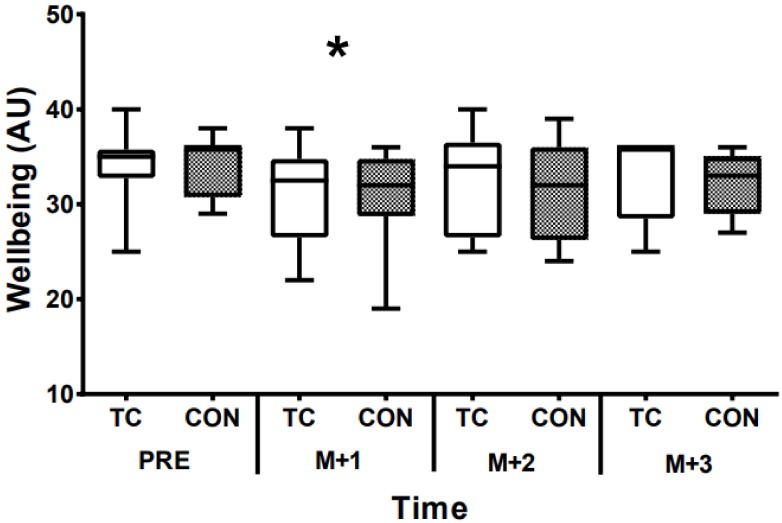
Wellbeing scores before (PRE) and three days (match day+1, match day+2, match-day+3 (M+1; M+2, M+3, respectively)) after a Rugby Union match and control (CON) or tart cherry juice (TC) supplementation. * Denotes different to PRE time point (*p* < 0.05) *n* = 10.

Baseline MS was 38.8 ± 29.3 mm in CON and 33.9 ± 25.1 mm in TC. MS increased at M+1 and M+2 but was similar to baseline levels by M+3 (time effect; *p* = 0.0001; ηp^2^ = 0.821; Figure 1). There were no differences between CON and TC at any time point (*p* = 0.807; ηp^2^ = 0.035). Wellness scores were lower at M+1 only (time effect; *p* = 0.023; ηp^2^ = 0.638), irrespective of supplementation (*p* = 0.647; ηp^2^ = 0.160; Figure 2). 

## 4. Discussion

We found that twice daily consumption of TC before and after a RU match did not attenuate MS or alter wellbeing in the three days following. To the author’s knowledge, this is the first study to suggest that supplementing with TC does not beneficially affect markers of recovery in professional athletes. 

Previous studies on the effects of TC for attenuating MS have been mixed and, thus, our findings are in agreement with some [11,17], but not the majority [3,6,7,8,18]. The reason for the discrepancy in findings in the studies conducted to date with TC is not entirely clear, given that similar doses, types of participants, exercise tasks, and measures of soreness have been used in studies showing positive or no effects of TC. It could be that studies without positive findings were not well powered enough to detect subtle differences in MS, given the inter-individual variation tends to be large, or that the magnitude of soreness was not great enough. The variations in cherry juice supplements and possibly phenolic contents could also help to explain the disparate findings to date; however, we are unaware of any published data addressing this. 

Nonetheless, there could be some specific reasons as to why we did not find any benefits in this trial compared to others. Perhaps the most likely reason is because the participants in the present study are elite athletes, whereas all the participants in previous studies showing beneficial effects were all sub-elite or recreationally active. As suggested with other functional foods, such as beetroot juice, perhaps elite athletes have exhausted almost all of their adaptive potential and are therefore less likely to benefit from additional supplementation [16,19]. In addition to this, it could also be because most other studies restricted dietary intake and all other recovery procedures following the muscle-damaging exercise protocols. However, the reality is that in elite sport, supplements are often taken concomitantly with others and diets are not restricted, questioning the ecological validity of these previous studies. 

Another possible explanation is that the TC doses were not big enough, given the body mass of the participants in this study was far greater than those in previous studies. As a result, the anti-inflammatory or antioxidant properties—which is the rationale for TC’s supplementation—were simply not great enough to significantly alter soreness levels. This is purely speculative at present, because we are not aware of studies that have assessed the bioavailability of these nutrients for different levels of body mass, but it would be reasonable to assume that those with higher body mass, especially lean mass, would require higher doses to get the same physiological effects—as is suggested with whey protein, for example [20]. This should be explored in future work. 

We hypothesized that TC might also alter wellbeing, because it has been shown to promote sleep [21] and reduce MS in some studies [3,6,7,8]. The lack of benefits in this study could be due to any of the explanations presented above, including the fact that the wellbeing questionnaire is not sensitive enough to detect subtle changes, given that it is un-validated and for in-house use. Ideally, we would have used a more sensitive measure such as muscle function and included inflammatory markers, to get a more holistic idea of the effects TC might be having on recovery; however, these were not possible in this population. We nonetheless recognize this as a limitation of the research and implore future research to try and collect this data when conducting research with elite athletes. 

A key limitation of this study is that we were only able to collect subjective markers of recovery after exercise, which are generally regarded as less valid then more objective markers of recovery. Indeed, previous studies have suggested that subjective MS does not necessarily correspond with changes in histological muscle damage or other indirect markers of such as muscle function and creatine kinase efflux and thus is a less valid predictor of overall recovery [1,22]. However, because of the elite nature of the population being studied, we were unable to take any additional measures as to not disrupt their daily training routines. We would also like to stress that because MS might heighten an individual’s risk of injury due to pain-related alterations in movement patterns during activity [23], and influence the degree of effort [13], we still believe its measurement provides useful information from a recovery perspective. It is also important to note that the subjective measurement of MS used in this study is also the same as that used in previous studies suggesting benefits of MS and, thus, our findings are no less valid than those previously reported. 

Another limitation of this study is the relatively low sample size. As a result, it is possible that we were underpowered to detect any subtle but meaningful differences between the two conditions, especially given the larger inter-individual variation observed with our primary outcome measure of MS. However, it is important to note that we did have a similar number of participants as in previous studies that examined TC and thus our sample size was no lower than the previous studies showing benefits of TC. Notwithstanding, future research should focus on recruiting higher participant numbers to increase their ability to detect subtle differences, if they exist. 

In conclusion, TC supplementation before and after a RU match did not attenuate muscle soreness or affect daily perceptions of wellbeing. Although these initial findings question whether TC is an effective recovery aid in real-world athletic situations, we stress that further research with different dosage protocols, additional measures of recovery, and stricter design controls are required before any definitive conclusions can be made on its efficacy. 

## Figures and Tables

**Table 1:** External load during 80 min of match-play for the two conditions (cherry-flavoured control gel (CON) vs. tart cherry juice (TC)). Total distance is the total distance covered during the match; high speed distance refers to the distance travelled accelerating at ≥5 m∙sec^−1^, sprint distance is the distance travelled at ≥70% of individualised maximum speed (km∙h^−1^); player load is the sum of all external load, including accelerations and decelerations. *n* = 10.

Variable	TC	CON	*p*-Value
Total distance (m)	6869.4 ± 1009.0	6822.0 ± 749.9	0.911
High speed distance (m)	530.5 ± 238.0	543.6 ± 245.6	0.798
Sprint distance (m)	82.1 ± 36.3	91.7 ± 39.4	0.506
Player load (AU)	712.7 ± 115.2	749.0 ± 126.7	0.542
